# Lateral variability of ichnological content in muddy contourites: Weak bottom currents affecting organisms’ behavior

**DOI:** 10.1038/s41598-019-54246-3

**Published:** 2019-11-27

**Authors:** Javier Dorador, Francisco J. Rodríguez-Tovar, Anxo Mena, Guillermo Francés

**Affiliations:** 1 Department of Earth Sciences, Royal Holloway University of London, Egham, UK; 2 Departamento de Estratigrafía y Paleontología, Universidad de Granada, Granada, Spain; 3 Departamento de Xeociencias Mariñas e O.T., Universidade de Vigo, Vigo, Spain

**Keywords:** Palaeontology, Sedimentology, Palaeoceanography

## Abstract

Although bioturbation is commonly recognized in contourites, only a few studies have analyzed the ichnological content of these deposits in detail. These studies have mainly focused on meso-scale bigradational sequence (a coarsening upward followed by a fining-upward sequence resulting from variations in current velocity). Here we present data from gravitational cores collected along the NW Iberian Margin showing systematic variation in ichnological content across proximal to distal depocenters within a large-scale elongated contourite drift. Data demonstrate that tracemakers’ behavior varies depending on the distance relative to the bottom current core. Trace fossils are already known to be a useful tool for studying of contouritic deposits and are even used as criterion for differentiating associated facies (e.g., turbidites, debrites), though not without controversy. We propose a mechanism by which the distance to the bottom current core exerts tangible influence on specific macro-benthic tracemaker communities in contourite deposits. This parameter itself reflects other bottom current features, such as hydrodynamic energy, grain size, nutrient transport, etc. Ichnological analysis can thus resolve cryptic features of contourite drift depositional settings.

## Introduction

The role of bottom currents in shaping deep-sea deposits (i.e., contourites) is currently a matter of debate in the scientific community^1,2^. Due to their implications for reconstruction of depositional conditions, contourite deposits have become a critical topic of investigation within the sub-disciplines of paleoceanography, slope-stability, and petroleum exploration. Due to their relative inaccessibility, however, contourites remain somewhat enigmatic. Only few studies have managed to investigate bioturbation and ichnofabrics in contour current settings^3–6^. Despite ongoing controversies^7,8^, trace fossil content is considered both a criterion for characterizing contouritic deposits and also a proxy for paleoenvironmental conditions^1,5,7^. These records are typically overprinted by bottom current activity^9^. In recent years, detailed ichnological studies conducted on contourite deposits in outcrops and core material have provided new insights into depositional processes, environmental conditions and the influence of bottom currents on tracemakers^5,6,10,11^. Due to lack of detailed records, the ichnological paradigm for contourites remains somewhat tentative. Here we describe unequivocal trace fossils in contouritic deposits from deep-sea gravity cores. Comparison of features reveals distinctive lateral variation with relative to bottom current cores.

**Figure 1:**
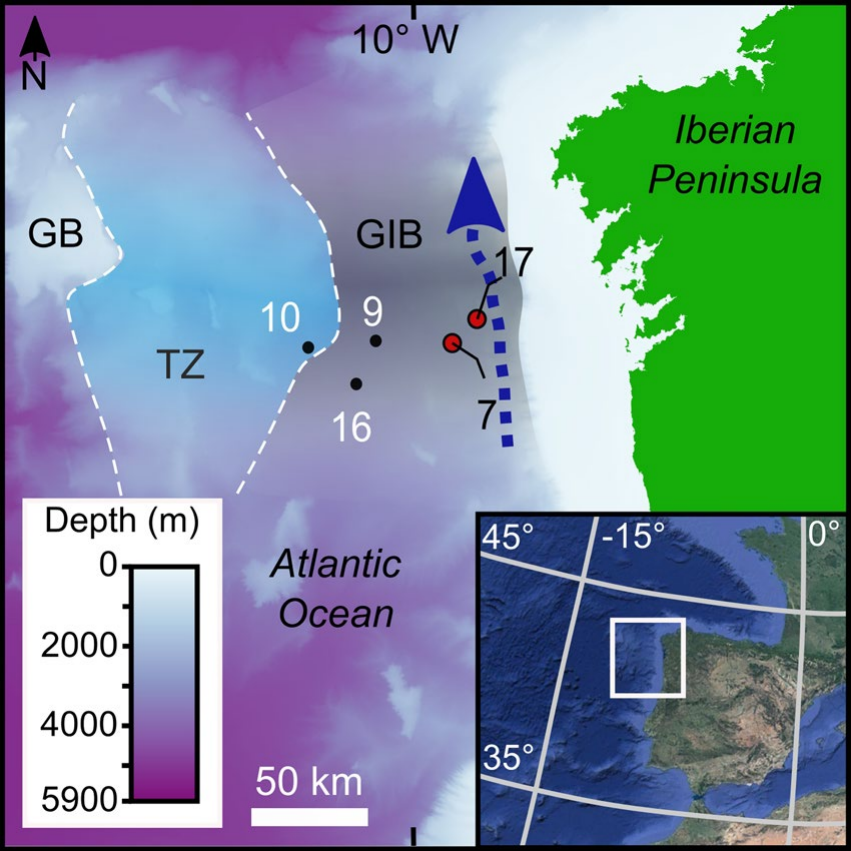
Location of cores analysed in this study. GB, Galicia Bank; GIB, Galicia Interior Basin; TZ, Transitional Zone. The arrow indicates the location of the bottom current (Mediterranean Outflow Water) during contourite deposition. The name of each gravity core sample begins with *FSG09-*. Inlay map from Google, Landsat/Copernicus. Globe image by Pixabay.

The present study investigated core material collected from about 3,000 m water depth^12^ during the ForSaGal 09 research cruise around the Galicia Interior Basin (GIB; Fig. 1). The location is known to be affected by northward bottom currents that interact with bathymetry to generate a contouritic drift along the basin. This setting provides a detailed record of Quaternary contourite deposits^13–16^. Contouritic facies appear as massive to coarsely laminated silt to very fine silty sand and, show varying degrees of bioturbation^17^.

### Ichnological content of contourites

**Figure 2:**
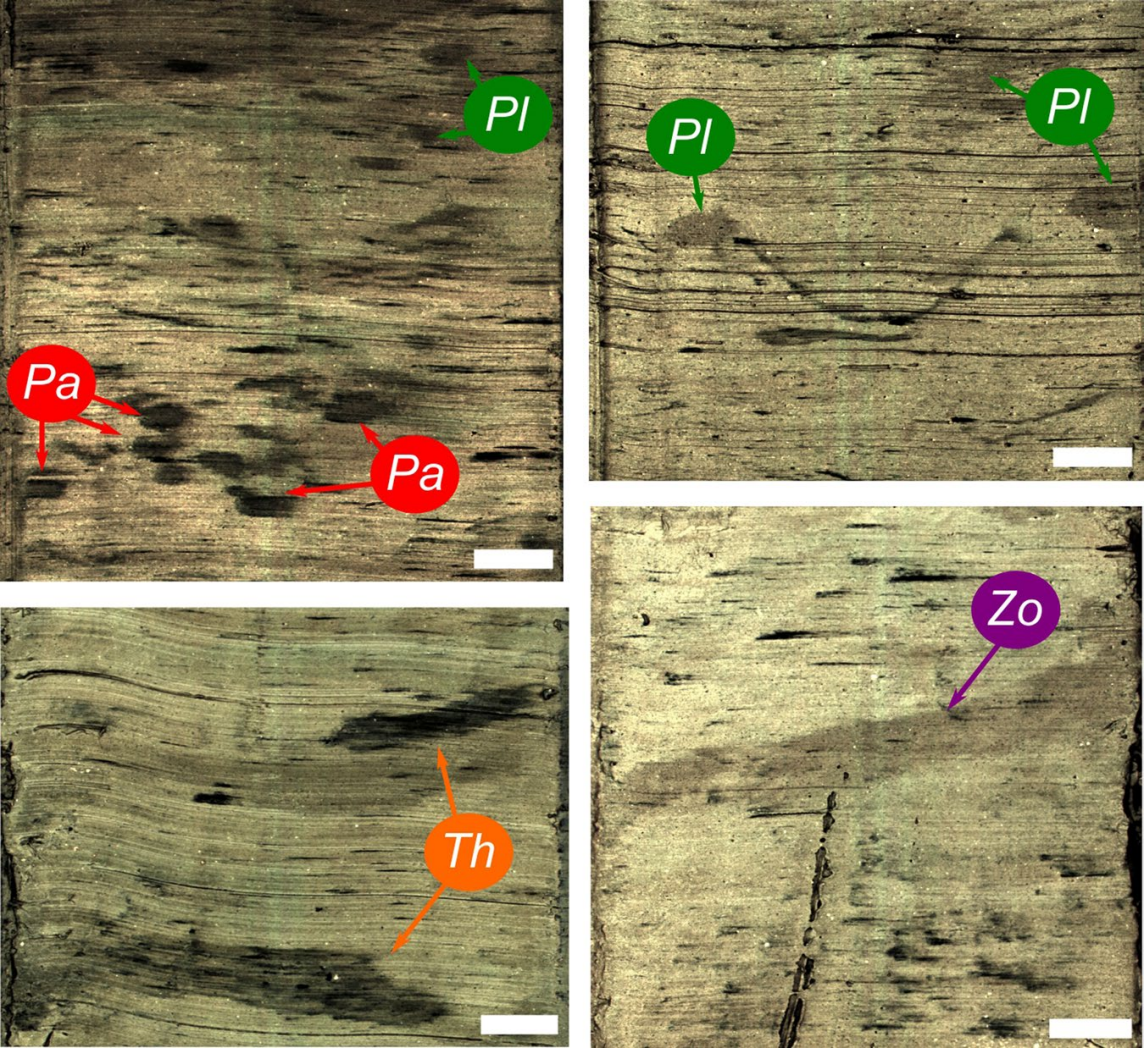
Ichnotaxa identified within contourite intervals. *Pa*, *Palaeophycus*; *Pl*, *Planolites*; *Th*, *Thalassinoides*; *Zo*, *Zoophycos*. Scale bar 1 cm. Apparent lamination is an artefact produced during core slabbing.

**Figure 3:**
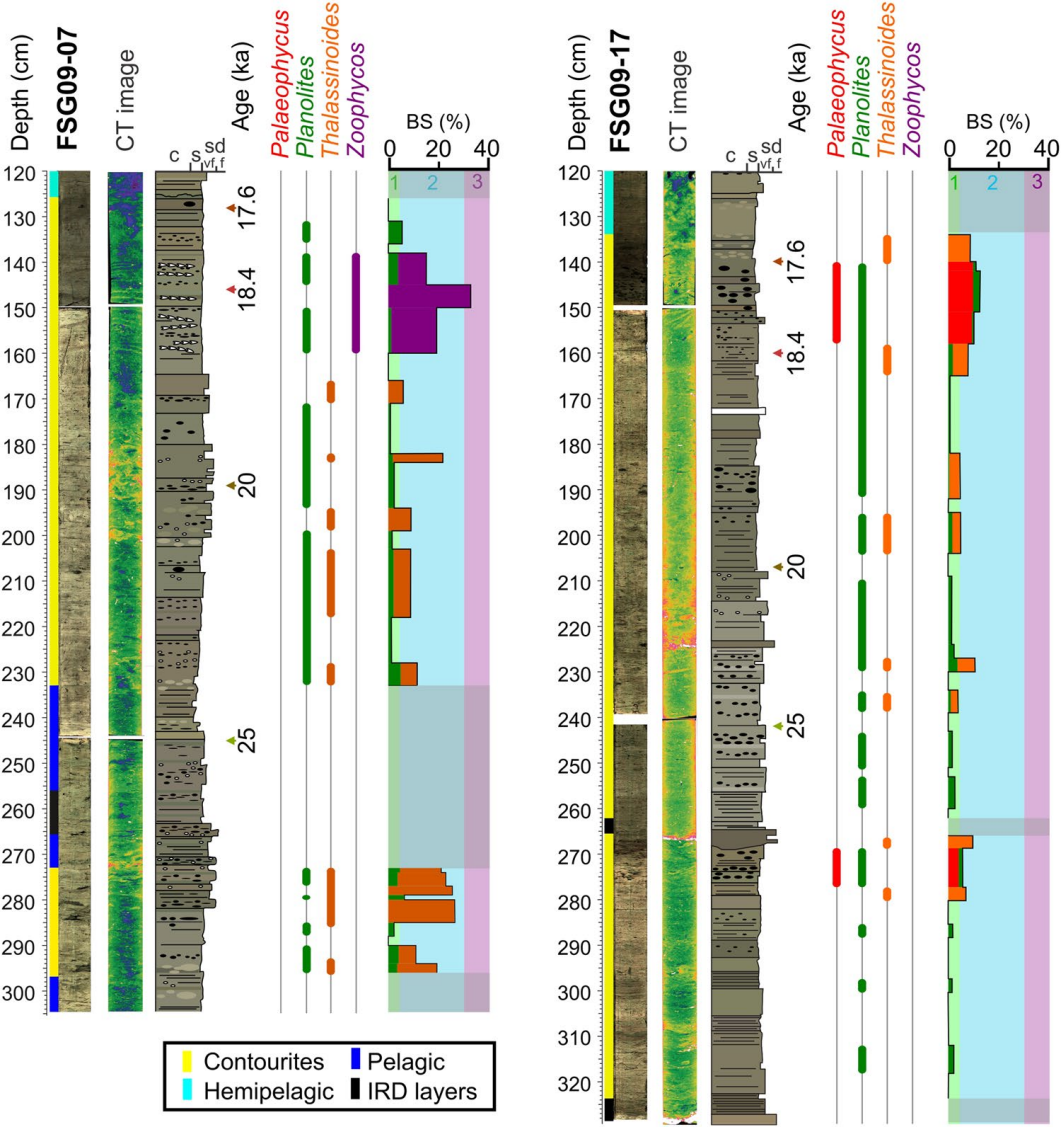
Sedimentological log and ichnologcial content of contourite deposits identified based on high-resolution images and CT data. BS: Bioturbated Surface.

Ichnological analysis of contouritic intervals from selected cores revealed an assemblage with relatively low diversity. In order of most to least dominant, contourite deposits contained *Thalassinoides*, *Planolites*, *Palaeophycus*, and *Zoophycos* (Fig. 2). *Thalassinoides* is defined as a 3D system of sub-horizontal burrows connected to the surface by sub-vertical shafts. Only limited sections of horizontal burrows appeared in cores analyzed by this study^18,19^. Burrows range from 4 to 18 mm in height and from 10 to 62 mm in length. *Planolites* are horizontal cylindrical tunnels, actively filled by the tracemaker^20^. These appear as sub-circular cross sections ranging from 3 to 15 mm in diameter. *Palaeophycus* are sub-horizontal cylindrical burrows characterized by passive filling and a lined wall^20,21^. These appear as lined sub-circular sections, 2–5 mm high and 4–19 mm long, filled by a darker sediment. Finally, *Zoophycos* is a complex helicoidal structure appearing as horizontal spreiten burrows in vertical sections of several cores^22,23^. The distribution of the ichnotaxa throughout the cores and their facies relationships (pelagic, hemipelagic, contourites and ice rafted debris or ‘IRD’) exhibit a clear pattern with contouritic intervals dominated by *Thalassinoides* and *Planolites* (Fig. 3). Abundant *Palaeophycus* or *Zoophycos* appear only occasionally (Fig. 3).

Bioturbated surfaces vary between 0 and 32% within contouritic intervals with a mean value of 6.8%. This value corresponds to a low to moderate BI (0 to 3) (Fig. 3). Contouritic intervals exhibit only minor bioturbation but they show a variable percentage of bioturbated surface, depending on the site. Core material from site FSG09-17 showed less bioturbation (3.7% bioturbated surface average) than that collected from site FSG09-07 (10.8% average) (Fig. 3). Every analyzed contouritic interval showed noteworthy vertical differences in percentages of bioturbated surface and in ichnological composition from bottom to top. At site FSG09-07 both contouritic intervals show increased bioturbation, which exceeded 30% and consist mainly of *Thalassinoides* at the bottom and *Zoophycos* at the top. The contouritic intervals from site FSG09-17 likewise record an increase in the bioturbated surface due to abundant *Palaeophycus* (Fig. 3).

### Ichnofabrics and paleoenvironmental conditions

The generally mottled background observed in contouritic core material reflects a complete reworking of the uppermost centimeters of the sediment. The degree of bioturbation in turn indicates relatively good environmental conditions for tracemakers working the unconsolidated substrate^24,25^. In this context, vertical changes in contourite ichnological features record significant variations in environmental parameters. Increasing percentages of bioturbated surface together with a shift in the dominant ichnotaxa reveal better conditions for particular tracemakers during the final stage of contourite deposition. Variation in *Zoophycos* in FSG09-07 and *Palaeophycus* in FSG09-17 indicates different ecological and depositional settings.

The organisms responsible for *Palaeophycus* constitute a eurybathic, facies-crossing ichnogenus, which occurs in a wide range of marine and non-marine settings. *Palaeophycus* itself represents a combined feeding/temporary dwelling burrow made by organisms with filter-, suspensive- and carnivore-feeding behaviors^20,26^. Their presence suggests high rates of organic matter transport to the sediment. *Palaeophycus* is a horizontal shallow burrow that is typically formed in shallow/middle tiers and remains open while the tracemakers live inside^20^. Dense occurrences of *Palaeophycus tubularis*, and the absence of other macroburrows, have been interpreted as evidence of ecologically stressful conditions during substrate colonization including salinity fluctuations, oxygen depletion, and turbidity. These features can also indicate a change in sedimentation rate or other conditions^26^.

*Zoophycos* is a deep tier structure with several ethological interpretations, but consensus favors the interpretation of cache behavior developed by vermiform animals^27,28^. A recent analysis describes relations between deep-marine *Zoophycos*, sedimentation rate, seasonal primary productivity, and oxygenation^29^. Accordingly, *Zoophycos* primarily appears in glacial periods with intensive seasonal productivity, reflecting high fluxes and intermediate sedimentation rates from 5 to 20 cm ka^−1^. Under these conditions, *Zoophycos* tracemakers collect nutrients at the sediment surface and transport them to deeper layers within the sediment to prevent oxidation^29^.

As outlined above, established interpretations of ichnological shifts attribute them to variations in sedimentation rate, organic matter availability, and oxygenation. Deposits from Galicia Interior Basin show clear spatiotemporal domains and associated sedimentation processes which themselves document the paleoenvironmental changes^12^. The westernmost FSG09-10 core was collected along the east flank of the Galicia Bank, in the so-called Transitional Zone^30^. This dome-like elevation is strongly influenced by bottom current activity that generates abraded surfaces^31^. Cores FSG09-09 and FSG09-16 were collected from the central part of the basin, between the Transitional Zone^31^ and lower slope^12,14^, which are dominated by pelagic and hemipelagic sedimentation^12^. Core FSG09-07, and especially the easternmost core FSG09-17, correspond to a contouritic and hemipelagic depositional setting developed on the lower continental slope^14^. Given their locations within the basin, sedimentation rate, organic matter availability and oxygenation vary with distance to the bottom current core.

**Figure 4:**
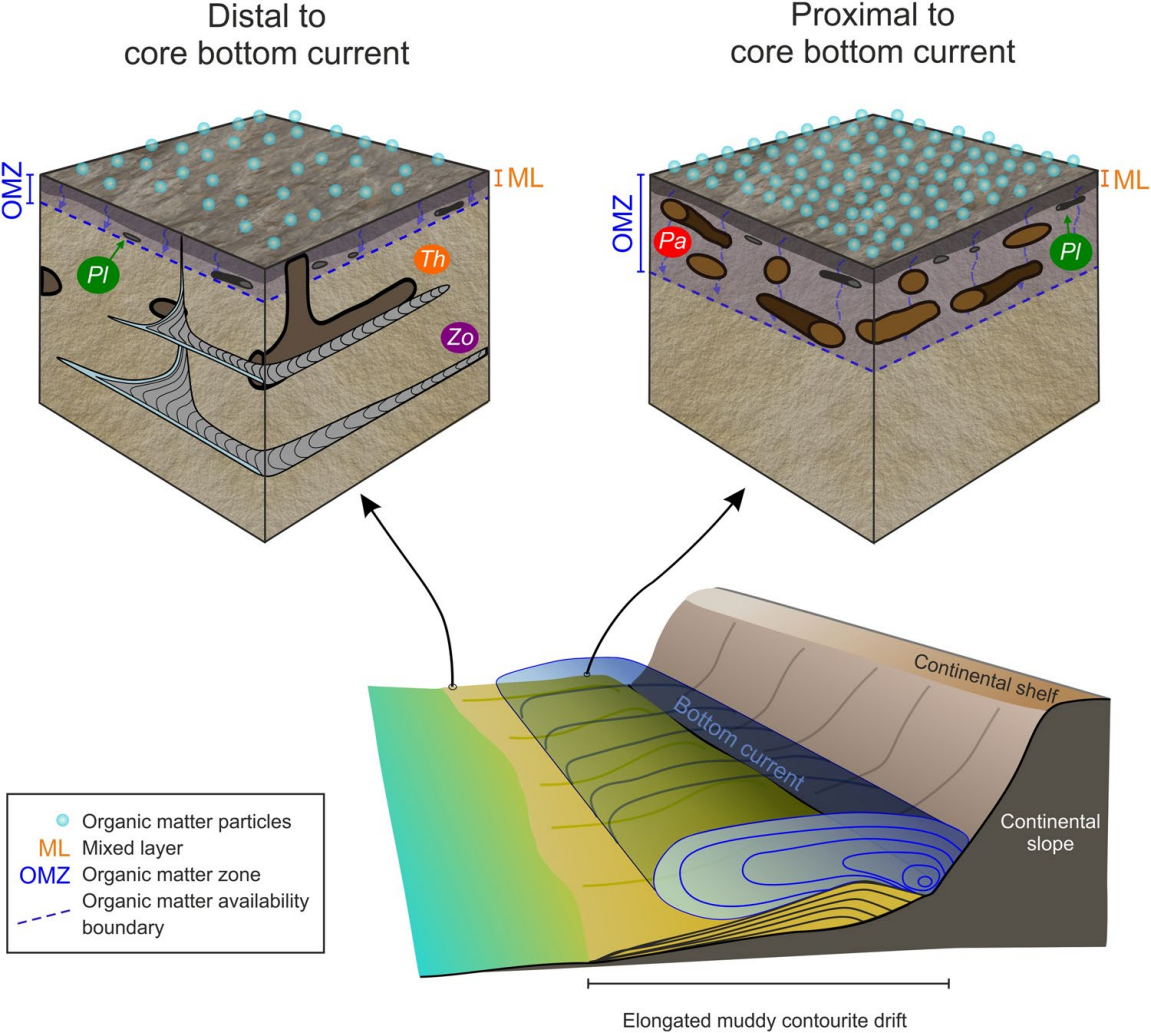
Schematic diagrams of ichnological features that characterize distal and proximal, to core bottom current, settings within a muddy contourite drift. *Pa*, *Palaeophycus*; *Pl*, *Planolites*; *Th*, *Thalassinoides*; *Zo*, *Zoophycos*.

Muddy contourites form due to the action of weak bottom currents that transport a substantial volume of organic matter particles^5,32,33^ which in turn feed benthic organisms^34,35^. Along the NW Iberian Margin for example, contour currents transport between 2–4 g m^−3^ of suspended material containing 40–100 mg m^−3^ of organic matter particles^33^. Distal zones to the core bottom current receive less sediment and organic matter than proximal settings, suggesting potential attendant variations in macrobenthic tracemaker communities. Site FSG09-17 (i.e., distal with respect to the bottom current core) experienced relatively low sedimentation rate and low organic matter flux. Organic matter at the sediment surface may be rapidly oxidized, preventing the development of shallow/middle tier structures. Under these conditions, only deep tier structures are produced, by organisms able to store organic matter deeper in the sediment as in the case of the *Zoophycos* tracemaker. Sediments from site FSG09-07 (i.e., the proximal site) indicate higher sedimentation rate and organic matter flux. Under these conditions, organic matter burial prevents oxidation and allows the development of shallow/middle tier dwelling structures, e.g. *Palaeophycus* (Fig. 4). The ichnological record, thus, systematically varies within the distal versus the proximal depositional zones,considering distance to core bottom current, of contourite drifts.

## Conclusions

Sedimentation rate, oxygen conditions, and organic matter availability influence the macrobenthic tracemaker communities and resulting trace fossil assemblage in actively forming contourite drifts. This report provides novel evidence of systematic proximal, to core bottom current, versus distal variation in ichnological features from muddy contourites. Varying depositional conditions are interpreted to reflect distance from the bottom current core. Sedimentation rate and organic matter availability are higher in proximal areas where organic matter is rapidly buried. This prevents oxidation and makes organic matter available for shallow tier tracemakers (e.g. *Palaeophycus* producers). In distal settings, sedimentation rate and organic matter availability is lower. Organic matter is rapidly oxidized at the surface, favoring development of middle and deep tier tracemakers, which transport organic matter to deeper layers of the sediment (e.g., *Zoophycos* producers). Systematic lateral variation in ichnological content of contourite drifts demonstrates an impactful role for ichnological analysis in contourite research. Trace fossils not only could differentiate contourites from turbidites and associated deposits, they also record proximal to distal deposition within a contourite drift relative to core bottom current features.

## Materials and Methods

Five gravity cores were collected during ForSaGal 09 in 2009 from the GIB, a narrow basin (around 100 km width; 2500–3000 m water depth) along the NW Iberian margin^17^. Sedimentary facies (i.e., Pelagic, Hemipelagic, Contouritic, Turbitidic and IRD layers) were previously characterized based on grain size, composition, sedimentary features and Computed Tomography (CT) scanning^12,17^. Contourite deposits, concretely muddy contourites, were identified in three cores. Only cores FSG09-07 (42.16°N, 9.84°W; 2393 mbsl) and FSG09-17 (42.26°N, 9.72°W; 2156 mbsl) (Fig. 1) contained a record long enough for detailed analysis. The present contribution used high resolution digital and CT images to perform ichnological analysis. Imaging steps used a CT scanner (HITACHI ECLOS 16 Multislice CT) at the Veterinary Teaching Hospital Rof Codina of Lugo (Galicia, Spain).

Ichnological analysis identified ichnotaxa based on ichnotaxabases (i.e., standard morphological features, wall, filling, etc.), tiering (i.e., vertical distribution of bioturbation structures within the sediment), crosscutting relations, and degree of bioturbation. The degree of bioturbation was determined by calculating the percentage of bioturbated surface using a quantification method based on high resolution digital images^36^, then calculated according to the Bioturbation Index scale^37,38^. These values reflect the spatial extent of discrete trace fossils identified over a common mottled background.

Age was calculated using the age model based on XRF data and AMS- ^14^C ages for the cores material analyzed^12^.

## Data Availability

All data analyzed during this study are summarized in this published article. The original datasets are not publicly available due to size restrictions but are available from the corresponding author by request.
